# A PCR-based quantitative assay for the evaluation of mRNA integrity in rat samples

**DOI:** 10.1016/j.bdq.2018.02.001

**Published:** 2018-03-16

**Authors:** Bhaja K. Padhi, Manjeet Singh, Marianela Rosales, Guillaume Pelletier, Sabit Cakmak

**Affiliations:** a Hazard Identification Division, Environmental Health Science and Research Bureau, Health Canada, Ottawa, Ontario, K1A 0K9, Canada; b Population Studies Division, Environmental Health Science and Research Bureau, Health Canada, Ottawa, Ontario, K1A 0K9, Canada

**Keywords:** RNA integrity, Real-time quantitative PCR, Housekeeping gene, *Pgk1*, Gene expression, Rat

## Abstract

Reverse Transcription quantitative real-time PCR (RT-qPCR) is applied to quantify gene transcript levels in a wide range of investigations. Proper assessment of RNA integrity is essential for reliable assessment of gene expression levels, as RNA molecules are acutely vulnerable to degradation. However, RNA quality control measures are still infrequently reported in rat toxicological studies, which impede proper evaluation of gene expression data reliability. The high operational cost of microfluidic capillary electrophoresis systems along with paucity of alternative methods for the quantitative assessment of rat RNA integrity constitute potential hurdles to the systematic implementation and reporting of RNA integrity assessment in rat studies. This manuscript describes the adaptation of an alternative RT-qPCR-based 3′:5′ assay as an additional option for the quantitative assessment of rat RNA integrity. Two PCR primer sets were designed on the 3′ and 5′ regions of a rat housekeeping gene to evaluate RNA integrity by measuring the relative expression (3′:5′ ratio) of these amplicons. The 3′:5′ ratios were then compared to Agilent Bioanalyzer’s RNA integrity number (RIN) for a wide range of RNA samples originating from different tissues, cultured cell lines and rat strains that were prepared freshly, stored for years at −80 °C, purchased commercially or intentionally degraded. The 3′:5′ ratios and RIN values presented similar assessment of RNA integrity status from intact to heavily degraded samples. Based on the LOWESS regression of this large comparison dataset, 3′:5′ ratio threshold criteria equivalent to RIN cut-off values can be proposed for the selection of RNA samples for RT-qPCR analyses. This qPCR-based assay is easy to implement, cost-effective, and provides a reliable quantification of RNA integrity to assist in the selection of rat RNA samples suitable for downstream RT-qPCR gene expression analyses.

## 1 Introduction

RT-qPCR is widely used to measure relative changes in gene transcript levels in order to assess biological responses associated with disease or toxicant/drug exposure, and to validate high throughput microarray and RNA-seq data [[1], [2], [3]]. RNA samples, the starting material for these studies, are acutely vulnerable to degradation. The use of degraded RNA samples can lead to unreliable gene expression data and hence, proper evaluation of RNA integrity is essential for reproducible results [[4], [5], [6], [7]].

Traditionally, RNA integrity was evaluated qualitatively by inspecting the intensities of the 28S and 18S ribosomal RNA (rRNA) bands following agarose gel electrophoresis. More recently, manufacturers have developed automated microfluidics-based electrophoretic systems that calculate a quantitative RNA quality score based on the analysis of digitalized electropherograms by proprietary algorithms [8]. The Agilent Bioanalyzer system, one of the best known microfluidics-based platforms, assigns RNA Integrity Number (RIN) values ranging from 1 to 10 to categorize the integrity of RNA samples [9]. RIN values above 8.0 indicate intact, high quality RNA samples, between 5.0 and 8.0 moderately degraded samples, and below 5.0 degraded samples [5,6]. The use of RNA samples presenting RIN values above 5.0 is typically recommended to ensure reliable quantification of gene expression by RT-qPCR [5,6].

As most gene expression studies target protein coding genes, RT-qPCR-based methods such as the 3′:5′ assays were proposed to evaluate messenger RNA (mRNA) integrity status [10]. This 3′:5′ approach is based on the measurement of the relative expression of two amplicons located on the 3′ and 5′ regions of a house-keeping gene transcript by RT-qPCR following cDNA synthesis using (anchored) oligo-dT primers [4,10,11]. In theory, reverse transcription should proceed uninterrupted in intact mRNA samples, generating similar levels of 3′ and 5′ amplicons resulting in a 3′:5′ ratio approaching 1.0. In a degraded RNA sample, the interruption of cDNA synthesis from the poly-A tail will lead to reduced levels of the cDNA template for the 5′ amplicon, resulting in higher 3′:5′ ratios. The recently published Differential Amplicon Assay (ΔAmp) is another approach to assess mRNA integrity that uses paired qPCR assays producing long and short amplicons from the same region of an mRNA [12].

Rat is a commonly used species for the assessment of chemical toxicity *in vivo* and *in vitro*. A recent literature survey revealed that more than half of rat toxicological studies using RT-qPCR do not describe RNA quality control measures and that only about one in five reported RNA integrity assessment by electrophoretic-based methods (Fig. S1). Although this lack of reporting does not necessarily imply the absence of appropriate RNA quality controls, such widespread omissions nevertheless impede the proper evaluation of the reliability of gene expression data in rat toxicological studies. While agarose gel electrophoresis requires large quantities of RNA and only allows a qualitative evaluation of RNA integrity, the more quantitative microfluidics-based platforms imply further operational costs and require additional equipment that may not be accessible to all laboratories. The development of a simple, affordable and easily implementable alternative method to quantitatively assess rat RNA integrity may facilitate adherence to RNA quality control measures and reporting in rat toxicological studies. The 3′:5′ assay originally developed for human and using probe-based Taqman dye possess many of these attributes and can be adapted to different species and fluorescent detection chemistries [10,11,13]. In order to expand the available options for RNA quality control in studies assessing rat gene expression, we adapted and optimized this 3′:5′ approach for rat RNA samples. Using a wide range of intact to heavily degraded rat RNA samples from different cell and tissue types, we then compared the 3′:5′ ratios obtained to the trusted microfluidic-based RIN values that delineate RNA sample’s suitability for down-stream RT-qPCR gene expression analyses.

## 2 Materials and methods

### 2.1 Rat C6, PC12 and CGC cell culture and tissue samples

All cells were grown at 37 °C in a humidified incubator containing 5% CO_2_ in cell culture media supplemented with 100 IU/ml penicillin + 100 μg/ml streptomycin. C6 glial cells from American Type Cell Culture (ATCC, Rockville, MD, USA), were cultured in F-12K medium containing 2.5% (v/v) foetal bovine serum (FBS) and 15% (v/v) horse serum. They were grown to confluence before RNA isolation. PC12 pheochromocytoma cells from ATCC were maintained in Dulbecco’s Modified Eagle’s Medium (DMEM) containing 5% (v/v) FBS, 10% (v/v) horse serum and 2 mM L-glutamine. Upon exposure to nerve growth factor (NGF), dividing PC12 cells differentiate by developing axon-like projections [14]. Freshly seeded PC12 cells were allowed to grow for one day and differentiation was initiated by the addition of 50 ng NGF/ml. Total RNA was isolated from dividing and differentiating PC12 cells one day after NGF treatment. Frozen primary Cerebellar Granule Cells (CGCs) from post-natal day 7 (PND7) rat brain purchased from QBM Cell Science (Ottawa, Ontario, Canada) were thawed and seeded at approximately 500,000 cells/well in polyD lysine coated six-well plates. CGCs were grown in Neurobasal A and B27 culture media (20 mM potassium chloride and 1 mM l-glutamine). The cell culture media was replaced by fresh media after one day in culture and total RNA was extracted on the fourth day of culture.

Developing rat brains were harvested from PND14 and PND21 Sprague-Dawley pups following decapitation without anesthesia. The hippocampi were dissected immediately from the brains, flash-frozen in liquid nitrogen and stored at −80 °C until RNA isolation. Further details about rat perinatal exposures, tissue harvesting and RNA extraction can be found in the original developmental neurotoxicity study [15]. Animals were handled following the Canadian Council on Animal Care guidelines and the experimental procedures were approved by Health Canada’s Institutional Animal Care Committee.

### 2.2 RNA extraction

C6, PC12 and CGC cultures were washed with 1× Phosphate-Buffered Saline (PBS) prior to RNA isolation. The cells were lysed directly on the culture dish using the lysis buffer provided in Qiagen’s RNeasy Mini Plus kit for total RNA isolation, and genomic DNA was removed using Qiagen’s gDNA Eliminator columns following the manufacturer’s protocols (Qiagen, Toronto, ON, Canada). Total RNA from juvenile rat hippocampus was extracted using TRIzol (Invitrogen, Burlington, ON, Canada), and further purified using Qiagen’s RNeasy Mini Plus kit and gDNA Eliminator columns following the manufacturer’s protocols. Total RNA samples from various juvenile and adult tissues and different rat strains (n = 34) were purchased from Zyagen (San Diego, CA, USA), see Table S2.

### 2.3 Evaluation of RNA purity and integrity

A Nanodrop 1000 spectrometer (Thermo Fisher Scientific, Ottawa, ON, Canada) was used to measure absorbance at 260 nm (A260) to evaluate RNA concentration. RNA purity was estimated using the A260/A280 ratio and only samples presenting a ratio greater than 1.8 were kept for further analyses. RNA integrity was assessed by an Agilent 2100 Bioanalyzer, using the Agilent RNA 6000 Nano Kit (Agilent Technologies, Mississauga, ON, Canada). The Solaris RNA Spike Control kit (Thermo Fisher Scientific, Cat# K-002200-C1-100) was used to assess the presence of inhibitors in a subset of RNA samples (Fig. S2). A PCR-based approach developed in-house [16] was used to assess gDNA contamination in RNA samples purchased from a commercial supplier. All the samples tested proved to be free from inhibitors and gDNA contamination.

### 2.4 PCR primer design

**Fig. 1:**
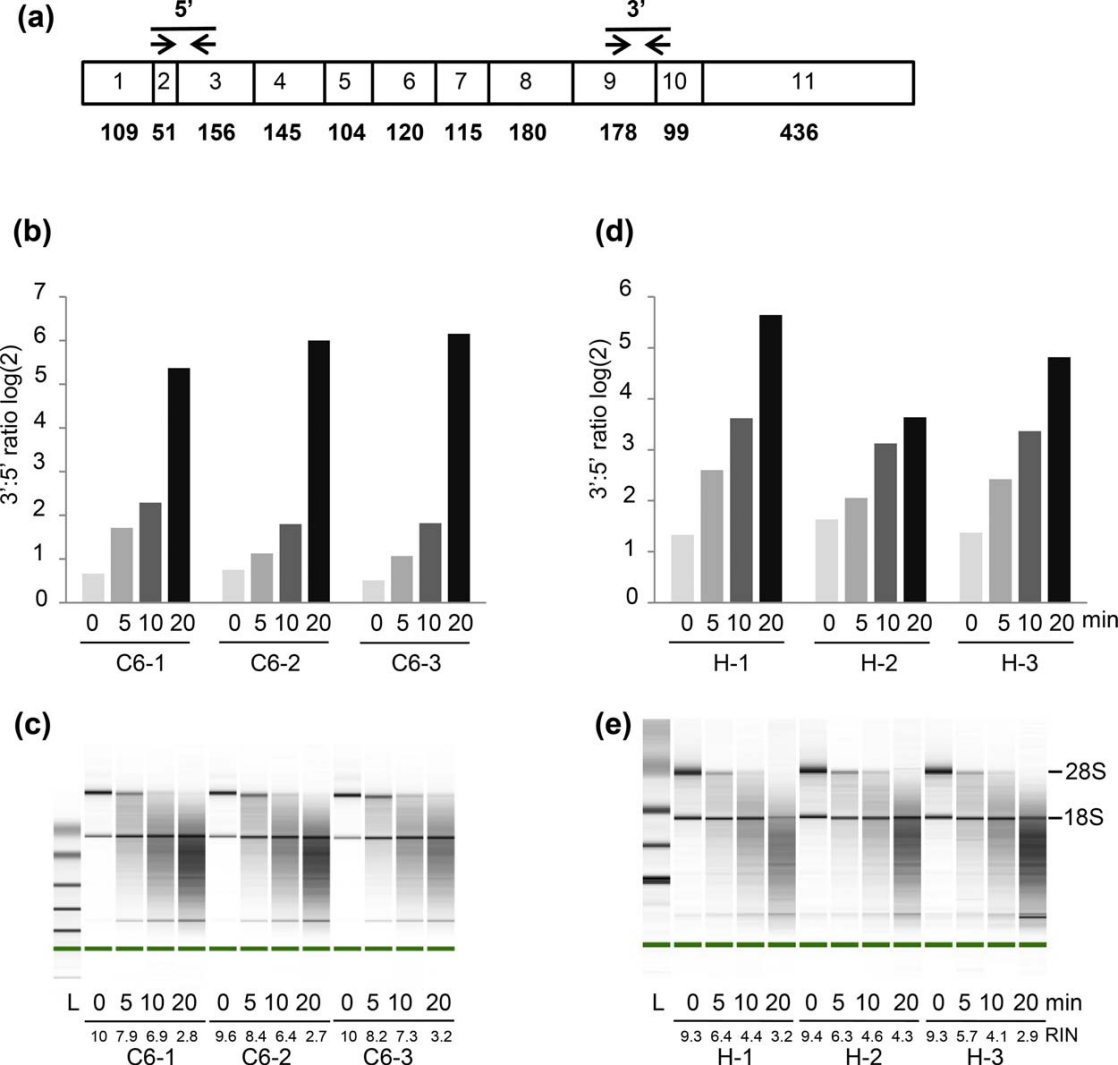
Development of the 3′:5′ assay. (a) Schematic representation of rat *Pgk1* mRNA showing the locations of the 5′ and 3′ amplicons. Exon boxes are numbered and their lengths in bp are indicated below. Three RNA samples from C6 cells (b–c) and PND 14 hippocampi (d–e) were heat-degraded (for 0, 5, 10 and 20 min at 90 °C) and then analyzed using the Agilent Bioanalyzer system and the proposed rat 3′:5′ assay. The intensities of 28S rRNA bands on Bioanalyzer’s electropherograms gradually decreased to completely disappear after 20 min of heat treatment (c and e). The 3′:5′ ratios gradually increased with RNA degradation, reaching maximal values of up to 71.2 (b) and 49.9 (d).

The ubiquitously expressed housekeeping gene *Phosphoglycerate kinase 1* (*Pgk1*, NM_053291) is well-suited for this 3′:5′ assay. The *Pgk1* gene possesses few pseudogenes and produces a relatively long transcript that presents a well-characterized exon-intron structure. While the low number of pseudogenes and exon-spanning primers will limit the potential interference from inadvertent genomic DNA contamination [16], the lengthy RNA sequence between the two amplified regions will likely contribute to the assay’s sensitivity to mRNA degradation. Two PCR primer sets spanning exon junctions and targeting the 3′ and 5′ regions of the *Pgk1* gene were designed (Fig. 1a, Table 1) using the web-based Primer3 software (www.bioinfo.ut.ee/primer3-0.4.0/primer3/input.htm). Primer–BLAST searches were conducted to check the specificity of these primer sets (https://www.ncbi.nlm.nih.gov/tools/primer-blast/). *In silico* PCR analyses of these primer sets at rat UCSC genome browser (genome assembly RGSC 6.0/rn6 at https://genome.ucsc.edu/cgi-bin/hgPcr) were performed to assess cross-match to any non-target sequence such as pseudogenes. The expected amplicon sequences were used to query the rat ENSEMBL database (http://useast.ensembl.org/Rattus_norvegicus/Info/Index) to ensure the absence of Single Nucleotide Polymorphisms (SNPs) at primer binding sites that would impair quantitative PCR efficiency [17]. The potential formation of secondary structures at the primer-template hybridization site that may interfere with PCR amplification was assessed by m-fold (http://mfold.rna.albany.edu/?q=mfold) [18]. The primers were synthesized at Eurofins genomics (Louisville, KY, USA).

**Table 1:** Sequences of the forward (FP) and reverse (RP) primers used for the rat 3′:5′ assay. Amplicon sizes, exonic locations, and nucleotide start and end points are also provided. Primer design was based on the 1685 nucleotides long sequence of the *Pgk1* gene (GenBank accession number NM_053291). The Rn-3′ amplicon is located 529 bp upstream of the 3′-end of *Pgk1* mRNA and is separated from the Rn-5′ amplicon by 856 bp.

Primer name	Primer sequence (5′-3′)	Amplicon size (bp)	Exonic location	Start	End
Rn-5′-FP	TCGTGATGAGGGTGGACTT	109	Exons 1–2	60	168
Rn-5′-RP	GCTCCATTGTCCAAGCAGA		Exon 3		

Rn-3′-FP	TGGGGTATTTGAATGGGAAG	107	Exon 9	1024	1130
Rn-3′-RP	TGTCTCCGCCTCCTATGATAGT		Exons 9–10		

### 2.5 cDNA synthesis, RT-PCR and RT-qPCR

The reverse transcription reactions were performed in a total volume of 20 μl using 0.5–2 μg of total RNA, 500 ng/μl Anchored Oligo-dT primers (Thermo Fisher Scientific), and 200 U Superscript III™ reverse transcriptase (Invitrogen) following manufacturer’s first-strand cDNA synthesis protocol. All cDNA samples were diluted in 9 volumes of sterile de-ionised water and stored at −20 °C until RT-qPCR analyses. Semi-quantitative RT-PCR reactions were carried out using PlatinumTaq (Invitrogen) in a C1000™ Thermal Cycler (Bio-Rad Laboratories, Hercules, CA, USA) for 35 cycles (30 s. denaturation at 94 °C followed by 30 s. annealing at 60 °C and 1 min extension at 72 °C). Quantitative PCR was performed with an iCycler iQ™5 Real-Time Detection System (Bio-Rad Laboratories) using 8 μl diluted cDNA, 0.4 μM of the forward (1 μl) and reverse (1 μl) primer, and 10 μl 2× SYBR-Green I dye master mix (QuantiTect^®^ SYBR^®^ Green PCR kit, Qiagen) in a reaction volume of 20 μl. The RT-qPCR reaction mix was denatured at 95 °C for 10 min and then subjected to 45 amplification cycles (10 s. denaturation at 95 °C, 45 s. annealing at 60 °C and 30 s. extension at 72 °C) [3]. Under these optimized qPCR conditions, each primer set presented amplification efficiency close to 100% (Fig. S4) and a single peak in melt-curve analyses.

### 2.6 Preparation of heat-degraded RNA samples

RNA samples from C6 and PC12 cell lines and PND14 hippocampi were diluted to 200 ng/μl. An aliquot (12 μl) of each diluted RNA sample was taken into four separate tubes. While one tube served as the control (0 min heat), the other three tubes were exposed to 90 °C heat for 5, 10 or 20 min in a thermocycler (Tables S1 and S2). For each tube, 1.0 μl of RNA sample was analyzed by Agilent Bioanalyzer, and 10 μl of RNA sample (2 μg) was used for cDNA synthesis as described above.

### 2.7 The 3′:5′ assay

PCR reactions for Rn-3′ and Rn-5′ were performed in duplicate on 96-well qPCR plates. In order to monitor for potential PCR artifacts, each plate also contained two no-template control wells (without cDNA), one for each primer set. RT-qPCR reactions were carried out in a total volume of 20 μl containing diluted cDNA (8 μl), 2 × SYBR-Green I dye (10 μl) and 0.4 μM of the appropriate *Pgk1* forward (1 μl) and reverse (1 μl) primers (Table 1), as described in Section 2.5. For each cDNA sample, the *Cq* values for the Rn-3′ and Rn-5′ amplicons were measured in duplicate. Technical replicates diverging by more than 0.5 *Cq* were excluded from the analysis and RT-qPCR reactions performed again. Technical replicate *Cq* values for Rn-3′ and Rn-5′ were averaged and used to calculate 3′:5′ ratios. Based on measured PCR efficiencies which were very close to 100%, the 3′:5′ ratios were calculated using the 2^(5′*Cq*−3′*Cq*)^ formula, based on Livak, & Schmittgen [19].

### 2.8 LOWESS analysis

LOcally WEighted Scatterplot Smoothing (LOWESS) regression [20] was applied to visualize the association between 3′:5′ ratios and RIN values. This robust non-parametric modeling approach was performed in R version 3.1.1. Based on this LOWESS regression and on the commonly accepted RIN cut-off value for the selection of suitable RNA samples for RT-qPCR, an equivalent 3′:5′ ratio threshold value was determined.

### 2.9 Concrete application of the proposed 3′:5′ assay and threshold ratio

High quality RNA samples from dividing (n = 3) and differentiating PC12 cells (n = 3) were subjected to heat degradation as described in Section 2.6. The integrity of intact and heat-treated RNA samples was assessed using both RIN values and 3′:5′ ratios (Table S2). The relative expression of *Transforming acidic coiled-coil containing protein 2* (*Tacc2)* was measured in intact, moderately degraded or highly degraded RNA samples following a previously described RT-qPCR experimental approach [3]. Two housekeeping genes (*Gapdh* and *Pgk1*) were used for the normalization of *Tacc2* expression, according to a modified ΔΔCq method [21]. The primer sequences of *Tacc2* [22], *Gapdh* [3] and *Pgk1* are presented in Table S3. Statistically significant differences in *Tacc2* mRNA abundance between dividing and differentiating PC12 cells were determined using a two-tailed Student’s *t*-test (p < 0.05).

## 3 Results

### 3.1 Development of 3′:5′ mRNA integrity assay

First, we selected a ubiquitously expressed and relatively long rat housekeeping gene (*Pgk1*), which also presents a well characterized intron-exon structure (Fig. 1a) and few pseudogenes. We designed two primer sets located far apart on the *Pgk1* transcript that spanned exon-exon boundaries and generated amplicons of a similar size (Table 1). PCR reactions for Rn-3′ and Rn-5′ were performed in separate tubes using intact brain RNAs (RIN > 8.0) from five different rat strains (Wistar, Lewis, Sprague-Dawley, Long-Evans and Fischer) and the specificity of primer sets for the 3′ and 5′ amplicons was assessed by endpoint RT-PCR. Amplification products from all rat strains produced a single band of the expected size on agarose gel (Fig. S3). The PCR amplicons were purified and sequenced, which further confirmed their identities. Both 3′ and 5′ primer sets produced a single peak in melt-curve analyses and presented very similar amplification efficiencies approaching 100% (Fig. S4) in an optimized RT-qPCR protocol based on the widely used SYBR-Green I dye.

Next, we assessed the ability of the proposed 3′:5′ ratio approach to identify degraded RNA samples. High-quality RNA samples (RIN > 9.3, A260/A280 ratio > 1.8) isolated in-house from C6 cells and PND14 hippocampus tissues were subjected to degradation by heat treatment at 90 °C for 0, 5, 10 and 20 min. These samples (n = 24) consisting of intact, moderately degraded and highly degraded RNAs (according to RIN values), were assessed using the 3′:5′ assay. As the level of RNA degradation in C6 and PND14 hippocampus samples increased, there was a corresponding decrease in RIN values (Fig. 1c, e), and increase in 3′:5′ ratios (Fig. 1b, d) (Table S1). These results clearly demonstrated that the proposed 3′:5′ assay can discriminate rat RNA samples presenting different degrees of degradation.

### 3.2 Comparison of 3′:5′ ratios and RIN values for a wide range of rat RNA samples

RNA samples isolated from 29 different tissues, three cell types and five rat strains were used to methodically compare 3′:5′ ratios to the commonly used RIN values. These RNA samples were freshly prepared, stored for years at −80 °C, experimentally- or accidentally-degraded, or purchased from a commercial supplier (Tables S1 and S2). High quality RNA (RIN > 9.8) prepared freshly from Cerebellar Granule Cells (CGCs) and PC12 cells, or C6 RNA samples stored at −80 °C for three years presented 3′:5′ ratios ranging from 1.4 to 2.5. Hippocampus RNA samples from PND21 rat pups stored at −80 °C for seven years showed RIN values between 7.7 and 8.7 and 3′:5′ ratios between 1.1 and 2.8. Of the 34 commercial RNA samples assessed, 21 tissues (heart, kidney, intestine, ovary, adipose, thyroid, spinal cord, hippocampus, pituitary, adrenal, skin thymus, adult brains from five different rat strains and developing brain at embryonic day 14, PND7 and PND14) showed RIN value >8.0, and their 3′:5′ ratio values ranged between 1.2–3.5. Another seven RNA samples (trachea, esophagus, pancreas, liver at PND1 and brain at PND1, PND21 and PND30) were moderately degraded (RIN 5.0–8.0) and presented 3′:5′ ratios ranging from 1.8 to 5.3. Accidental degradation of the remaining six RNA samples (cerebella at PND7, PND14 and PND30, blood, lung, and thalamus) during their transport resulted in low RIN values (2.5–4.8) and high 3′:5′ ratios (5.2–199.5).

### 3.3 Determination of 3′:5′ ratio threshold value for RNA sample selection

**Fig. 2:**
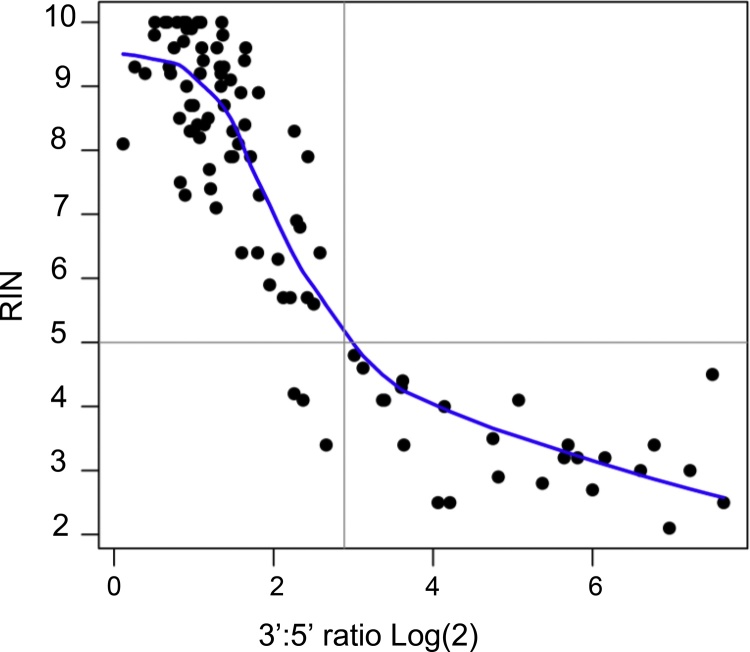
Comparison of 3′:5′ ratios and RIN values for a panel of 99 rat RNA samples. LOWESS regression shows a clear association between 3′:5′ ratios and RIN values for the RNA samples assessed (listed in Tables S1 and S2).

RIN values and 3′:5′ ratios for all the RNA samples tested (listed in Tables S1 and S2) were plotted on a graph and a non-parametric LOWESS regression was performed to better visualise the relationship between these two metrics (Fig. 2). As the measured RIN values for RNA samples decreased from 10 to 5.0, the 3′:5′ ratios gradually increased to reach a value of approximately 8.0 (Fig. 2). Thereafter, degraded RNA samples presented rapidly increasing 3′:5′ ratios, which reached values up to 200. Hence, based on this LOWESS regression and on the commonly accepted RIN cut-off value of 5.0 for the selection of suitable RNA samples for RT-qPCR analyses [[5], [6]], we propose an equivalent 3′:5′ ratio threshold of 8.0. The classifications of the RNA samples according to the RIN cut-off value and the newly proposed 3′:5′ ratio threshold were concordant for 96 of the 99 samples assessed in Fig. 2.

### 3.4 Concrete application of the 3′:5′ assay

**Fig. 3:**
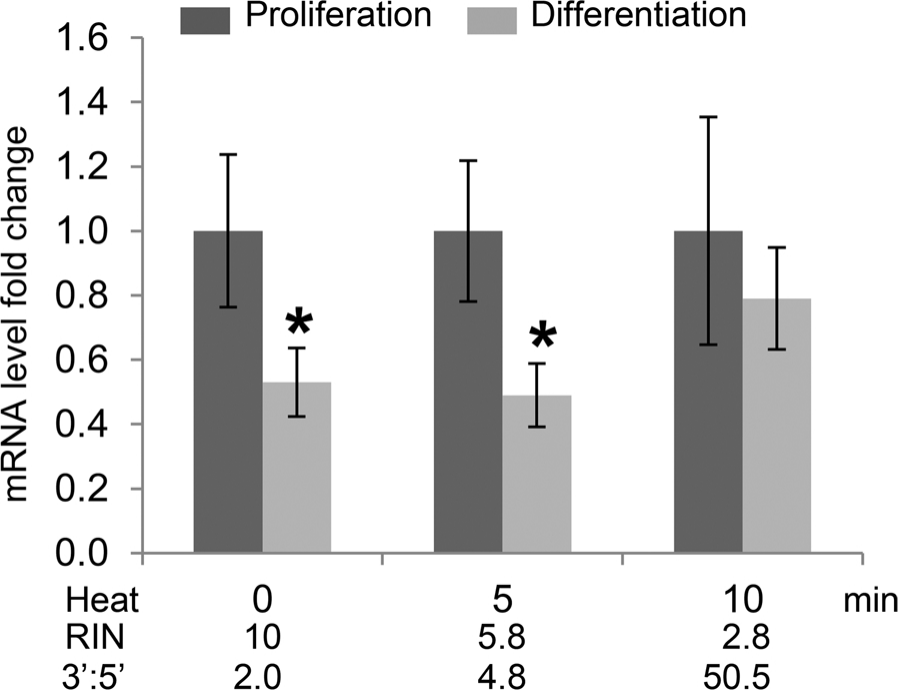
Measurement of *Tacc2* relative expression in proliferating (n = 3) and differentiating (n = 3) PC12 cells using intact (0 min heat), moderately degraded (5 min heat) or degraded (10 min heat) RNA samples. Average RIN and 3′:5′ ratio values are provided below each heat-treatment condition. Error bars represent standard error of the mean and * indicates a statistically significant difference (p < 0.05) according to two-tailed Student’s *t*-test.

PC12 cells proliferate in culture, but upon exposure to nerve growth factor (NGF) these cells exit the mitotic cycle and begin to differentiate by developing axon-like projections [14]. Given that *Tacc2* is involved in the organization of centrosomal tubules in proliferating cells [22], we hypothesized that its relative expression may decrease when proliferating PC12 cells undergo differentiation. To provide a concrete example of the application of the proposed 3′:5′ assay (and 3′:5′ ratio threshold), high quality RNA samples isolated from proliferating and differentiating PC12 cells were heated for 0, 5 and 10 min at 90 °C, to generate intact, moderately degraded and degraded RNA samples, according to RIN values and 3′:5′ ratios (Table S2). As expected, *Tacc2* relative expression was significantly lower in differentiating PC12 cells, when assessed using intact or moderately degraded RNA samples (3′:5′ ratio <8.0) (Fig. 3). However, the differential expression of *Tacc2* between proliferating and differentiating PC12 cells was no longer statistically significant when assessed using degraded RNA samples presenting 3′:5′ ratios above the proposed threshold value (Fig. 3). These experiments further illustrate the importance of proper RNA quality controls for reliable measurements of gene expression.

## 4 Discussion

Our survey of RT-qPCR-based gene expression analyses in rat toxicological studies (Fig. S1) clearly supports the claims that RNA quality controls generally receive insufficient attention [1,23]. Systematic reporting of RNA integrity assessment would allow for a better evaluation of gene expression data reliability in rat toxicological studies, as RNA degradation adversely affects the reproducibility of RT-qPCR data [[5], [6],^9^]. Given that microfluidic-based electrophoresis systems may not be universally accessible in laboratories performing rat *in vivo* or *in vitro* toxicology studies, the development of an alternative option for rat RNA integrity assessment may contribute to improved monitoring and reporting of RNA quality controls. The 3′:5′ assay is one such option, but this approach had not yet been adapted for the rat species.

Although the 3′:5′ assay is conceptually simple, numerous technical aspects still needed to be carefully considered for its adaptation to rat RNA samples. We selected a ubiquitously expressed and relatively long rat housekeeping gene (*Pgk1*), which also presents a well characterized intron-exon structure. We then designed primer sets located far apart on the *Pgk1* transcript that spanned exon-exon boundaries and generated amplicons of a similar size (Table 1). The DNase treatment of RNA samples and the selection of a gene presenting few pseudogenes further limited the potential effects of inadvertent genomic contamination [16]. These primer sets (Rn-3′ and Rn-5′) presented similar PCR amplification efficiencies (Fig. S4) and we confirmed their specificities by sequencing the amplicons and by melt-curve analyses in RT-qPCR. The cDNA amplification was detected by SYBR-Green I, a widely used intercalating fluorescent dye that is compatible with all RT-qPCR systems, further facilitating adoption of the assay by any laboratory measuring rat gene expression. All RT-qPCR reactions were performed in duplicate. Technical replicates presenting *Cq* values varying by less than 0.5 were averaged and used to calculate 3′:5′ ratios.

As a next-step towards the development of a rat 3′:5′ assay, we tested the ability of the proposed protocol to discriminate between intact and heat-degraded RNA samples (Fig. 1). As expected, increasingly degraded RNA samples presented a progressive reduction of 28S rRNA band intensity and RIN values, as 3′:5′ ratios increased. Although the 3′:5′ assay is based on a single gene that may not reflect the integrity status of all types of mRNA transcripts, this investigation suggested that the 3′:5′ assay can provide an assessment at least roughly similar to microfluidic-based electrophoresis methods that mainly use rRNA degradation as a proxy for mRNA integrity [24].

Then, we compared 3′:5′ ratios to the widely-used RIN values for a wide range of rat RNA samples from different cells, tissues and rat strains that also presented varying levels of degradation (Table S1 and S2). These 3′:5′ ratios and RIN values plotted in Fig. 2 were somewhat scattered, but overall presented an easily discernible association. This scattering may be explained by the fact that while the RIN values are derived from the electrophoretic profiles of total RNA, the 3′:5′ ratios also assess the efficiency and processivity of the reverse transcription used to synthesize cDNA.

For the rat 3′:5′ assay to be used in a concrete experimental setting, it was critically important to empirically determine practical 3′:5′ ratio thresholds to guide the selection of RNA samples suitable for downstream applications. Based on the LOWESS regression of the scatterplot presented in Fig. 2, we derived a 3′:5′ ratio threshold of 8.0 which corresponds to a RIN value of 5.0, the lower boundary for moderately degraded RNA [[5], [6]]. Incidentally, Bio-Rad’s human and mouse 3′:5′ assays also suggested a similar Δ*Cq* threshold of 3.0 [13], which corresponds to our 3′:5 ratio of 8.0 (2^3^ = 8). Discrepancy with the 3′:5′ ratio threshold of 5.0 proposed by Nolan et al. [10], may be explained by differences in the selected gene, PCR primer design, PCR conditions or fluorescent probe chemistry. Further, the LOWESS regression presented in Fig. 2 can be used to establish 3′:5′ ratio threshold corresponding to any desired RIN value. For example, a RIN value of 7.0 would correspond to a 3′:5′ ratio of 4.0. Although 3′:5′ ratio thresholds have been proposed for other 3′:5′ assays [10,11,13], to our knowledge, this investigation represents the most extensive benchmarking of a 3′:5′ assay against a trusted microfluidic-based electrophoresis method published so far.

Finally, we compared *Tacc2* relative expression in proliferating and differentiating PC12 cells using intact or degraded RNA samples (Fig. 3). Although RT-qPCR assays may tolerate a certain level of RNA degradation when small amplicon (<250 bp) and proper normalization strategy are used [25], the differential *Tacc2* expression observed in intact to moderately degraded RNA was no longer statistically significant in severely degraded RNA samples (3′:5′ ratio >8.0, and RIN <5.0).

This concrete example of the application of the 3′:5′ assay to the measurement of *Tacc2* gene expression is yet another reminder of the importance of proper RNA quality controls for reliable and reproducible RT-qPCR analyses. However, for studies specifically attempting to measure gene expression in RNA samples presenting moderate to severe enzymatic degradation (from post-mortem samples for example), further characterization of the 3′:5′ assay may be needed. Contrary to heat treatment, enzymatic-mediated RNA degradation is a non-random process initiated by either 3′-end polyA tail/5′-cap removal, followed by exonucleolytic decay or endonucleolytic cleavage [8]. Consequently, additional comparison of 3′:5′ ratios and RIN values for enzymatically degraded RNA samples will be required before applying this method to investigations that specifically attempt to assess gene expression from RNA samples presenting significant enzymatic degradation.

## 5 Conclusions

This study describes the adaptation of a 3′:5′ RNA integrity assay for the rat species. Comparison of 3′:5′ ratios and RIN values for a wide range of rat RNA samples revealed a good association between these two RNA integrity metrics. Based on RIN cut-off values for the selection of RNA samples for downstream RT-qPCR analyses, this extensive comparison allows the empirical determination of equivalent 3′:5′ ratio thresholds. For the vast majority of samples assessed, both methods produced concordant categorization of RNA sample suitability for RT-qPCR applications. Finally, we provided a concrete example of the application of the 3′:5′ assay in an experimental protocol, which constitutes yet another reminder of the importance of proper RNA quality controls. Based on these results, we conclude that the described 3′:5′ assay, which can be easily performed in any laboratory measuring rat gene expression by RT-qPCR, represents a valid alternative option for the quantitative evaluation of RNA integrity.

## Competing financial interests

The authors declare no competing financial interests.
