# Supplementary material for: A PCR-based quantitative assay for the evaluation of mRNA integrity in rat samples
Source: Biomol Detect Quantif. 2018 Mar 16;15:18–23. doi: 10.1016/j.bdq.2018.02.001 (PMC6006387; doi:10.1016/j.bdq.2018.02.001)
Supplement: Supplementary file 1 [file mmc1.docx]

**Appendix A: Supplementary data**

**A PCR-based quantitative assay for the evaluation of mRNA integrity in rat samples**

Bhaja K. Padhi^1^, Manjeet Singh^1^, Marianela Rosales^1^, Guillaume Pelletier^1^, Sabit Cakmak^2^

^1^Hazard Identification Division, ^2^Population Studies Division, Environmental Health Science and Research Bureau, HECSB, Health Canada, Tunney’s Pasture, Ottawa, Ontario, K1A 0K9, Canada

**Index of Tables and Figures:**

**Table S1:** Development of the 3':5' assay for rat RNA samples using *Pgk1* Rn-3'and Rn-5' amplicons after heat treatment at 90 ºC for 0, 5, 10 and 20 minutes.

**Table S2:** Comparative assessment of 3’:5’ ratios and RIN values in various rat RNA samples.

**Table S3:** Sequences of the PCR primers used to assess the impact of RNA degradation on *Tacc2* transcript

levels.

**Figure S1:** A survey of RNA quality control measures reported in rat toxicological studies.

**Figure S2:** Assessment of contaminating qPCR inhibitors using the Solaris RNA Spike Control kit.

**Figure S3:** RT-PCR amplification of *Pgk1* target sequences in various rat strains.

**Figure S4:** Amplification efficiencies of the Rn-3’ and Rn-5’ *Pgk1* primer sets.

**Table S1:**

**Development of the 3':5' assay for rat RNA samples using *Pgk1* Rn-3'and Rn-5' amplicons after heat treatment at 90 ºC for 0, 5, 10 and 20 minutes.***

|  |  |  |  |  |  |  |  |
| --- | --- | --- | --- | --- | --- | --- | --- |
|  | **RNA Sample** | **Heat** | **Rn-3' *Cq*** | **Rn- 5' *Cq*** | **∆*Cq*** | **3':5' ratio** | **RIN** |
| 1 | C6 S#1 | 0 min | 18.32 | 18.99 | 0.666 | 1.587 | 10 |
| 2 | C6 S#2 | 0 min | 18.79 | 19.54 | 0.751 | 1.683 | 9.6 |
| 3 | C6 S#3 | 0 min | 19.36 | 19.88 | 0.513 | 1.427 | 10 |
| 4 | C6 S#1 | 5 min | 17.91 | 19.62 | 1.711 | 3.275 | 7.9 |
| 5 | C6 S#2 | 5 min | 18.18 | 19.31 | 1.133 | 2.192 | 8.4 |
| 6 | C6 S#3 | 5 min | 18.91 | 19.98 | 1.071 | 2.101 | 8.2 |
| 7 | C6 S#1 | 10 min | 19.24 | 21.52 | 2.287 | 4.882 | 6.9 |
| 8 | C6 S#2 | 10 min | 19.15 | 20.95 | 1.800 | 3.482 | 6.4 |
| 9 | C6 S#3 | 10 min | 19.06 | 20.89 | 1.821 | 3.532 | 7.3 |
| 10 | C6 S#1 | 20 min | 19.91 | 25.28 | 5.37 | 41.322 | 2.8 |
| 11 | C6 S#2 | 20 min | 21.05 | 27.05 | 6.00 | 63.885 | 2.7 |
| 12 | C6 S#3 | 20 min | 20.97 | 27.12 | 6.15 | 71.169 | 3.2 |
| 13 | Hippocampus PND14 S#1 | 0 min | 16.94 | 18.26 | 1.33 | 2.511 | 9.3 |
| 14 | Hippocampus PND14 S#2 | 0 min | 17.23 | 18.87 | 1.64 | 3.107 | 9.4 |
| 15 | Hippocampus PND14 S#3 | 0 min | 16.91 | 18.28 | 1.38 | 2.594 | 9.3 |
| 16 | Hippocampus PND14 S#1 | 5 min | 17.13 | 18.73 | 1.60 | 3.031 | 6.4 |
| 17 | Hippocampus PND14 S#2 | 5 min | 17.46 | 19.52 | 2.05 | 4.155 | 6.3 |
| 18 | Hippocampus PND14 S#3 | 5 min | 17.29 | 19.71 | 2.42 | 5.353 | 5.7 |
| 19 | Hippocampus PND14 S#1 | 10 min | 18.56 | 22.18 | 3.62 | 12.275 | 4.4 |
| 20 | Hippocampus PND14 S#2 | 10 min | 18.26 | 21.39 | 3.12 | 8.703 | 4.6 |
| 21 | Hippocampus PND14 S#3 | 10 min | 18.13 | 21.49 | 3.36 | 10.296 | 4.1 |
| 22 | Hippocampus PND14 S#1 | 20 min | 19.02 | 24.66 | 5.64 | 49.880 | 3.2 |
| 23 | Hippocampus PND14 S#2 | 20 min | 19.36 | 22.99 | 3.63 | 12.402 | 3.4 |
| 24 | Hippocampus PND14 S#3 | 20 min | 19.12 | 23.93 | 4.82 | 28.177 | 2.9 |

* C6 RNA samples were extracted from freshly grown cells. Hippocampus RNA samples were extracted seven years earlier and stored at ultra-cold temperature in DEPC-treated water.

**Table S2:**

**Comparative assessment of 3’:5’ ratios and RIN values in various rat RNA samples.***

|  | **RNA source** | **RNA Sample Status** | **Rn-3' *Cq*** | **Rn-5' *Cq*** | **∆Cq** | **3':5' ratio** | **RIN** |
| --- | --- | --- | --- | --- | --- | --- | --- |
|  | **A. In-house samples** |  |  |  |  |  |  |
|  |  |  |  |  |  |  |  |
| 1 | C6 cells S#1 | Stored 2 years at -80 ºC | 14.42 | 14.93 | 0.507 | 1.421 | 9.8 |
| 2 | C6 cells S#2 | Stored 2 years at -80 ºC | 14.50 | 15.42 | 0.914 | 1.884 | 9.9 |
| 3 | C6 cells S#3 | Stored 2 years at -80 ºC | 14.85 | 15.75 | 0.904 | 1.871 | 10 |
| 4 | C6 cells S#4 | Stored 2 years at -80 ºC | 14.68 | 15.47 | 0.790 | 1.729 | 10 |
| 5 | C6 cells S#5 | Stored 2 years at -80 ºC | 14.51 | 15.15 | 0.639 | 1.558 | 10 |
| 6 | C6 cells S#6 | Stored 2 years at -80 ºC | 14.80 | 15.70 | 0.902 | 1.869 | 10 |
|  |  |  |  |  |  |  |  |
| 7 | Primary Cerebellar Granule | Fresh RNA | 17.11 | 18.47 | 1.36 | 2.571 | 9.8 |
|  | Cells after 4 days of culture |  |  |  |  |  |  |
|  |  |  |  |  |  |  |  |
| 8 | PC12 48 hrs CON #1 | Fresh RNA | 14.56 | 15.43 | 0.87 | 1.828 | 10 |
| 9 | PC12 48 hrs CON #2 | Fresh RNA | 14.48 | 15.83 | 1.35 | 2.549 | 10 |
| 10 | PC12 48 hrs CON #3 | Fresh RNA | 15.16 | 16.21 | 1.05 | 2.071 | 10 |
|  |  |  |  |  |  |  |  |
| 11 | PC12 48 hrs NGF #1 | Fresh RNA | 15.16 | 16.05 | 0.89 | 1.853 | 10 |
| 12 | PC12 48 hrs NGF #2 | Fresh RNA | 14.53 | 15.5 | 0.97 | 1.959 | 9.9 |
| 13 | PC12 48 hrs NGF #3 | Fresh RNA | 14.82 | 15.91 | 1.09 | 2.129 | 10 |
|  |  |  |  |  |  |  |  |
| 14 | PC12 48 hrs CON #1 | 5 min heat treatment | 14.28 | 16.23 | 1.95 | 3.865 | 5.9 |
| 15 | PC12 48 hrs CON #2 | 5 min heat treatment | 13.88 | 16.39 | 2.50 | 5.669 | 5.6 |
| 16 | PC12 48 hrs CON #3 | 5 min heat treatment | 14.19 | 16.45 | 2.26 | 4.784 | 4.2 |
|  |  |  |  |  |  |  |  |
| 17 | PC12 48 hrs NGF #1 | 5 min heat treatment | 17.17 | 19.75 | 2.58 | 5.980 | 6.4 |
| 18 | PC12 48 hrs NGF #2 | 5 min heat treatment | 17.45 | 19.57 | 2.12 | 4.350 | 5.7 |
| 19 | PC12 48 hrs NGF #3 | 5 min heat treatment | 17.35 | 19.68 | 2.33 | 5.030 | 6.8 |
|  |  |  |  |  |  |  |  |
| 20 | PC12 48 hrs CON #1 | 10 min heat treatment | 16.87 | 20.26 | 3.39 | 10.483 | 4.1 |
| 21 | PC12 48 hrs CON #2 | 10 min heat treatment | 17.79 | 22.86 | 5.07 | 33.591 | 4.1 |
| 22 | PC12 48 hrs CON #3 | 10 min heat treatment | 17.33 | 21.47 | 4.14 | 17.630 | 4.0 |
|  |  |  |  |  |  |  |  |
| 23 | PC12 48 hrs NGF #1 | 10 min heat treatment | 17.47 | 21.07 | 3.60 | 12.126 | 3.3 |
| 24 | PC12 48 hrs NGF #2 | 10 min heat treatment | 17.84 | 22.59 | 4.75 | 26.909 | 3.5 |
| 25 | PC12 48 hrs NGF #3 | 10 min heat treatment | 19.53 | 27.17 | 7.64 | 199.466 | 2.5 |
|  |  |  |  |  |  |  |  |
| 26 | PC12 48 hrs CON #1 | 20 min heat treatment | 17.36 | 24.32 | 6.96 | 124.500 | 2.1 |
| 27 | PC12 48 hrs CON #2 | 20 min heat treatment | 17.06 | 23.66 | 6.60 | 97.006 | 3.0 |
| 28 | PC12 48 hrs CON #3 | 20 min heat treatment | 18.77 | 25.99 | 7.22 | 149.086 | 3.0 |
|  |  |  |  |  |  |  |  |
| 29 | PC12 48 hrs NGF #1 | 20 min heat treatment | 16.55 | 23.32 | 6.77 | 109.137 | 3.4 |
| 30 | PC12 48 hrs NGF #2 | 20 min heat treatment | 16.81 | 22.5 | 5.69 | 51.625 | 3.4 |
| 31 | PC12 48 hrs NGF #3 | 20 min heat treatment | 17.09 | 22.9 | 5.81 | 56.103 | 3.2 |
|  |  |  |  |  |  |  |  |
| 32 | hippocampus PND21 CON#1 | Stored 7 years at -80 ºC | 15.57 | 16.39 | 0.820 | 1.766 | 8.5 |
| 33 | hippocampus PND21 CON#2 | Stored 7 years at -80 ºC | 15.91 | 16.95 | 1.045 | 2.063 | 8.4 |
| 34 | hippocampus PND21 CON#3 | Stored 7 years at -80 ºC | 15.42 | 16.42 | 0.996 | 1.994 | 8.3 |
| 35 | hippocampus PND21 CON#4 | Stored 7 years at -80 ºC | 15.83 | 17.32 | 1.491 | 2.811 | 7.9 |
| 36 | hippocampus PND21 CON#5 | Stored 7 years at -80 ºC | 15.62 | 16.82 | 1.195 | 2.290 | 7.7 |
|  |  |  |  |  |  |  |  |
| 37 | hippocampus PND21 OC#1 | Stored 7 years at -80 ºC | 15.72 | 16.90 | 1.181 | 2.267 | 8.5 |
| 38 | hippocampus PND21 OC#2 | Stored 7 years at -80 ºC | 16.39 | 16.50 | 0.112 | 1.081 | 8.1 |
| 39 | hippocampus PND21 OC#3 | Stored 7 years at -80 ºC | 15.42 | 16.90 | 1.488 | 2.805 | 8.3 |
| 40 | hippocampus PND21 OC#4 | Stored 7 years at -80 ºC | 15.92 | 16.87 | 0.952 | 1.935 | 8.3 |
| 41 | hippocampus PND21 OC#5 | Stored 7 years at -80 ºC | 15.49 | 16.49 | 0.996 | 1.995 | 8.7 |
|  |  |  |  |  |  |  |  |
|  | **B. Commercial samples** |  |  |  |  |  |  |
|  |  |  |  |  |  |  |  |
|  | (a) Adult tissues |  |  |  |  |  |  |
| 42 | heart | Stored 6 months at -80 ºC | 16.8 | 17.06 | 0.26 | 1.197 | 9.3 |
| 43 | kidney | Stored 6 months at -80 ºC | 17.62 | 18.01 | 0.39 | 1.310 | 9.2 |
| 44 | intestine | Stored 6 months at -80 ºC | 17.69 | 18.81 | 1.12 | 2.173 | 9.4 |
| 45 | ovary | Stored 6 months at -80 ºC | 17.43 | 18.14 | 0.71 | 1.636 | 9.2 |
| 46 | liver | Stored 6 months at -80 ºC | 17.33 | 18.43 | 1.10 | 2.144 | 9.6 |
| 47 | adipose | Stored 6 months at -80 ºC | 17.84 | 18.53 | 0.69 | 1.613 | 9.3 |
| 48 | thyroid | Stored 6 months at -80 ºC | 18.08 | 19.73 | 1.65 | 3.138 | 9.6 |
| 49 | pituitary | Stored 6 months at -80 ºC | 18.38 | 19.84 | 1.46 | 2.751 | 9.1 |
| 50 | spinal cord | Stored 6 months at -80 ºC | 16.86 | 18.15 | 1.29 | 2.445 | 9.6 |
| 51 | hippocampus | Stored 6 months at -80 ºC | 17.00 | 18.59 | 1.59 | 3.010 | 8.9 |
| 52 | adrenal | Stored 6 months at -80 ºC | 15.97 | 16.93 | 0.96 | 1.945 | 8.7 |
| 53 | skin | Stored 6 months at -80 ºC | 17.91 | 20.17 | 2.26 | 4.790 | 8.3 |
| 54 | thymus | Stored 6 months at -80 ºC | 17.56 | 18.94 | 1.38 | 2.603 | 8.7 |
| 55 | trachea | Stored 6 months at -80 ºC | 20.24 | 22.67 | 2.43 | 5.389 | 7.9 |
| 56 | esophagus | Stored 6 months at -80 ºC | 16.94 | 17.77 | 0.83 | 1.778 | 7.5 |
| 57 | pancreas | Stored 6 months at -80 ºC | 25.5 | 27.71 | 2.21 | 4.627 | 5.7 |
| 58 | lung | Stored 6 months at -80 ºC | 22.10 | 29.6 | 7.50 | 181.019 | 4.5 |
| 59 | blood | Stored 6 months at -80 ºC | 22.25 | 25.26 | 3.01 | 8.056 | 4.8 |
| 60 | thalamus | Stored 6 months at -80 ºC | 17.42 | 19.79 | 2.37 | 5.169 | 4.1 |
|  |  |  |  |  |  |  |  |
|  | (b) Developing tissues |  |  |  |  |  |  |
| 61 | liver PND1 | Stored 6 months at -80 ºC | 17.69 | 19.15 | 1.46 | 2.751 | 7.9 |
| 62 | brain embryonic day 20 | Stored 6 months at -80 ºC | 15.87 | 16.74 | 0.87 | 1.828 | 9.7 |
| 63 | whole brain PND1 | Stored 6 months at -80 ºC | 17.58 | 18.79 | 1.21 | 2.313 | 7.4 |
| 64 | whole brain PND7 | Stored 6 months at -80 ºC | 17.73 | 19.07 | 1.34 | 2.532 | 9.0 |
| 65 | whole brain PND14 | Stored 6 months at -80 ºC | 15.45 | 16.53 | 1.08 | 2.114 | 9.2 |
| 66 | whole brain PND21 | Stored 6 months at -80 ºC | 15.42 | 16.31 | 0.89 | 1.853 | 7.3 |
| 67 | whole brain PND30 | Stored 6 months at -80 ºC | 15.91 | 17.19 | 1.28 | 2.428 | 7.1 |
| 68 | cerebellum PND7 | Stored 6 months at -80 ºC | 18.72 | 21.38 | 2.66 | 6.320 | 3.4 |
| 69 | cerebellum PND14 | Stored 6 months at -80 ºC | 18.92 | 22.98 | 4.06 | 16.679 | 2.5 |
| 70 | Cerebellum PND30 | Stored 6 months at -80 ºC | 19.1 | 23.31 | 4.21 | 18.507 | 2.5 |
|  |  |  |  |  |  |  |  |
|  | (c) Rat strains |  |  |  |  |  |  |
| 71 | Wistar (adult brain) | Stored 6 months at -80 ºC | 15.99 | 16.9 | 0.91 | 1.879 | 9.0 |
| 72 | Sprague-Dawley (adult brain) | Stored 6 months at -80 ºC | 15.70 | 17.26 | 1.56 | 2.949 | 8.1 |
| 73 | Lewis (adult brain) | Stored 6 months at -80 ºC | 15.54 | 17.35 | 1.81 | 3.506 | 8.9 |
| 74 | Fischer-344 (adult brain) | Stored 6 months at -80 ºC | 17.05 | 18.69 | 1.64 | 3.117 | 8.4 |
| 75 | Long-Evans (adult brain) | Stored 6 months at -80 ºC | 16.83 | 18.17 | 1.34 | 2.532 | 9.2 |

* RNA samples were stored at -80 ºC. Abbreviation used: CON - Control (no treatment); NGF - Nerve Growth Factor (inducer of PC12 differentiation); OC - Organochlorine pesticides (perinatal exposure), PND - Post-Natal Day.

**Table S3:**

**Sequences of the PCR primers used to assess the impact of RNA degradation on *Tacc2* transcript levels.**

| **Gene name** | **Accession** | **Primer sequence**  **(5'-3')** | **Amplicon size (bp)** | **Location** |
| --- | --- | --- | --- | --- |
| *Tacc2* | NM_001004418  NM_001004415 | F-AAAGAACGCTGGAGCAGAAG  R-GACGGTCGTATTGCACACAA | 134 | Exon 21  Exon 22 |
| *Pgk1* | NM_053291 | F-TGGGAACAAGGTTAAAGCTGA  R-CTCTGTGTGCAGTCCCAAAA | 107 | Exons 4-5  Exon 5 |
| *Gapdh* | NM_017008.3 | F-AGTTCAACGGCACAGTCAAG  R-CATACTCAGCACCAGCATCAC | 120 | Exon 3  Exon 4 |

***A survey of RNA quality control measures reported in rat toxicological studies (Fig S1)***

**Figure S1:** A survey of RNA quality control measures reported in rat toxicological studies. Reporting of RNA purity (absence of contaminating inhibitory substance, protein, DNA) and integrity (intactness/degradation) before RT-qPCR assessment of gene expression in rat toxicological studies was surveyed in research articles from four reputable toxicology journals (Impact Factor > 3, see the references below). Manuscripts were retrieved from the journal websites using the key words “real-time PCR” and “rat”. One hundred suitable research articles published between January 2010 and December 2016 were randomly selected. The reporting of RNA purity and integrity assessment was categorised as follows: i) spectrophotometry; ii) agarose gel electrophoresis; iii) spectrophotometry plus microfluidics-based methods; iv) spectrophotometry plus agarose gel electrophoresis; and v) not mentioned. This survey revealed that more than half (52%) of the sampled research articles did not report RNA quality controls and only 22% reported performing RNA integrity assessment, either by agarose gel electrophoresis or by microfluidics methods.

***The qPCR inhibitor assay (Fig S2)***

To ensure that the RNA samples used in this study were free from inhibitors that may impact qPCR efficiency, we used the Solaris RNA Spike Control kit (Thermo Fisher Scientific, Cat# K-002200-C1-100) following manufacturer's instructions (<https://static.thermoscientific.com/images/D19882~.pdf>). The RNA spike control is a synthetic nucleic acid sequence lacking homology to mammalian genome sequence. Briefly, 2 μl of 10× Solaris Spike solution was added to 2 μg of RNA samples (from C6 cells, liver or PND21 hippocampus) or to PCR grade RNase-free water in a final volume of 20 μl. Reverse transcription was then carried out and cDNA samples to be used for RT-qPCR were then diluted 10 times. The RT-qPCR reactions were conducted in a 20 μl volume which contained diluted cDNA (8 μl), 20× Solaris qPCR spike assay (1 μl), water (1 μl), and the 2× Solaris qPCR Master mix (10 μl) including the primers (Forward primer, 5’-TGCAAAGCCAATTCCCGAAG-3’; Reverse primer, 5’-CCATTGTAGTGAACAGTAGGAC-3’) required for the amplification of the synthetic RNA sequence. The *Cq* values of total rat RNA samples spiked with synthetic RNA measured in an iQ5 system real-time PCR machine were compared to those of the control reactions (synthetic RNA in PCR grade water). In principle, the presence of inhibitors in the tested samples should result in higher *Cq* values than those observed in the control reactions. The absence of inhibitors is assumed for ∆*Cq* < 2, likely inhibition is inferred for ∆*Cq* between 2 and 3, and definite inhibition for ∆*Cq* > 3.

*
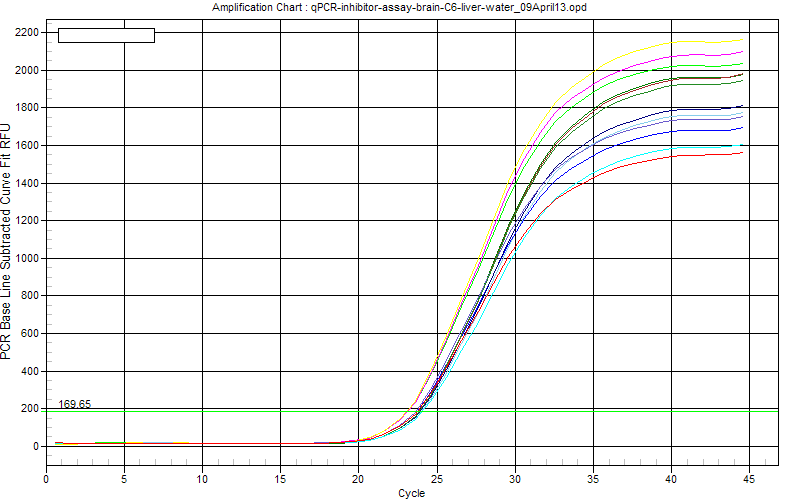
*

**Figure S2:** Assessment of contaminating qPCR inhibitors using the Solaris RNA Spike Control kit. RNA samples from PND21 hippocampus and C6 cells were isolated in-house, while liver RNA sample was purchased from a commercial source. The *Cq* values for RNA samples did not significantly differ from those of the water-spiked controls (∆*Cq* < 1), suggesting an absence of inhibitors that may impact qPCR reactions.

***Specificity of the Rn-5’ and Rn-3’ primer sets in different rat strains (Fig S3)***


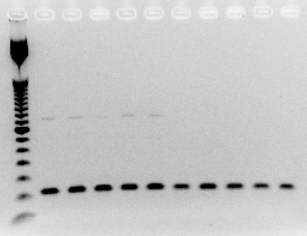


**Rn-5’ Rn-3’**

**50 bp ladder**

**Wistar**

**Lewis**

**Sprague-Dawley**

**Long-Evans**

**Fischer**

**Wistar**

**Lewis**

**Sprague-Dawley**

**Long-Evans**

**Fischer**

**Figure S3:** RT-PCR amplification of *Pgk1* target sequences in various rat strains. Adult brain RNA samples from five different rat strains all generated a single band following RT-PCR amplifications using Rn-5’ and Rn-3’ primer sets.

***Primer efficiency test (Fig S4)***


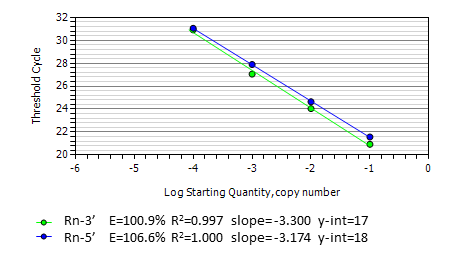


**Figure S4:** Amplification efficiencies of the Rn-3’ and Rn-5’ *Pgk1* primer sets.

**References**

[1] H. Abe, F. Saito, T. Tanaka, S. Mizukami, Y. Watanabe, N. Imatanaka, Y. Akahori, T. Yoshida, M. Shibutani, Global gene expression profiles in brain regions reflecting abnormal neuronal and glial functions targeting myelin sheaths after 28-day exposure to cuprizone in rats. Toxicol.Appl.Pharmacol. 310 (2016) 20-31.

[2] M. Adler, D. Hoffmann, H. Ellinger-Ziegelbauer, P. Hewitt, K. Matheis, L. Mulrane, W.M. Gallagher, J.J. Callanan, L. Suter, M.M. Fountoulakis, W. Dekant, A. Mally, Assessment of candidate biomarkers of drug-induced hepatobiliary injury in preclinical toxicity studies. Toxicol.Lett. 196 (2010) 1-11.

[3] C.A. Aiub, G. Gadermaier, I. Oliveira, I. Felzenszwalb, F. Ferreira, L.F. Ribeiro Pinto, P. Eckl, N-Nitrosodiethylamine genotoxicity in primary rat hepatocytes: effects of cytochrome P450 induction by phenobarbital. Toxicol.Lett. 206 (2011) 139-143.

[4] M.A. Ansari, Z.H. Maayah, S.A. Bakheet, A.O. El-Kadi, H.M. Korashy, The role of aryl hydrocarbon receptor signaling pathway in cardiotoxicity of acute lead intoxication in vivo and in vitro rat model. Toxicology 306 (2013) 40-49.

[5] I. Baranowska-Bosiacka, L. Struzynska, I. Gutowska, A. Machalinska, A. Kolasa, P. Klos, G.A. Czapski, M. Kurzawski, A. Prokopowicz, M. Marchlewicz, K. Safranow, B. Machalinski, B. Wiszniewska, D. Chlubek, Perinatal exposure to lead induces morphological, ultrastructural and molecular alterations in the hippocampus. Toxicology 303 (2013) 187-200.

[6] E.B. Bian, C. Huang, H. Wang, X.X. Chen, L. Zhang, X.W. Lv, J. Li, Repression of Smad7 mediated by DNMT1 determines hepatic stellate cell activation and liver fibrosis in rats. Toxicol.Lett. 224 (2014) 175-185.

[7] A.J. Blanca, M.V. Ruiz-Armenta, S. Zambrano, J.L. Miguel-Carrasco, J.L. Arias, M. Arevalo, A. Mate, O. Aramburu, C.M. Vazquez, Inflammatory and fibrotic processes are involved in the cardiotoxic effect of sunitinib: Protective role of L-carnitine. Toxicol.Lett. 241 (2016) 9-18.

[8] J. Blanco, M. Mulero, L. Heredia, A. Pujol, J.L. Domingo, D.J. Sanchez, Perinatal exposure to BDE-99 causes learning disorders and decreases serum thyroid hormone levels and BDNF gene expression in hippocampus in rat offspring. Toxicology 308 (2013) 122-128.

[9] P.C. Boutros, C.Q. Yao, J.D. Watson, A.H. Wu, I.D. Moffat, S.D. Prokopec, A.B. Smith, A.B. Okey, R. Pohjanvirta, Hepatic transcriptomic responses to TCDD in dioxin-sensitive and dioxin-resistant rats during the onset of toxicity. Toxicol.Appl.Pharmacol. 251 (2011) 119-129.

[10] S. Brandner, C. Eberhagen, J. Lichtmannegger, L. Hieber, U. Andrae, TCDD induces the expression of insulin-like growth factor binding protein 4 in 5L rat hepatoma cells: a cautionary tale of the use of this cell line in studies on dioxin toxicity. Toxicology 309 (2013) 107-116.

[11] L. Camacho, J.R. Latendresse, L. Muskhelishvili, R. Patton, J.F. Bowyer, M. Thomas, D.R. Doerge, Effects of acrylamide exposure on serum hormones, gene expression, cell proliferation, and histopathology in male reproductive tissues of Fischer 344 rats. Toxicol.Lett. 211 (2012) 135-143.

[12] G.M. Camerino, B.M. De, E. Conte, A. Liantonio, K. Musaraj, M. Cannone, A. Fonzino, A. Giustino, L.A. De, R. Romano, C. Camerino, A. Laghezza, F. Loiodice, J.F. Desaphy, C.D. Conte, S. Pierno, Statin-induced myotoxicity is exacerbated by aging: A biophysical and molecular biology study in rats treated with atorvastatin. Toxicol.Appl.Pharmacol. 306 (2016) 36-46.

[13] A. Caride, A. Lafuente, T. Cabaleiro, Endosulfan effects on pituitary hormone and both nitrosative and oxidative stress in pubertal male rats. Toxicol.Lett. 197 (2010) 106-112.

[14] T. Chen, W. Yang, Y. Li, X. Chen, S. Xu, Mono-(2-ethylhexyl) phthalate impairs neurodevelopment: inhibition of proliferation and promotion of differentiation in PC12 cells. Toxicol.Lett. 201 (2011) 34-41.

[15] J. Demenesku, A.A. Popov, I. Mirkov, M. Ninkov, L. Zolotarevski, D. Kataranovski, I. Brceski, M. Kataranovski, Strain differences of cadmium-induced toxicity in rats: Insight from spleen and lung immune responses. Toxicol.Lett. 256 (2016) 33-43.

[16] H. Dracinska, F. Barta, K. Levova, A. Hudecova, M. Moserova, H.H. Schmeiser, K. Kopka, E. Frei, V.M. Arlt, M. Stiborova, Induction of cytochromes P450 1A1 and 1A2 suppresses formation of DNA adducts by carcinogenic aristolochic acid I in rats in vivo. Toxicology 344-346 (2016) 7-18.

[17] A.Y. Efremenko, J.L. Campbell, Jr., D.E. Dodd, A.R. Oller, H.J. Clewell, III, Time- and concentration-dependent genomic responses of the rat airway to inhaled nickel subsulfide. Toxicol.Appl.Pharmacol. 279 (2014) 441-454.

[18] C.J. Ek, A. Wong, S.A. Liddelow, P.A. Johansson, K.M. Dziegielewska, N.R. Saunders, Efflux mechanisms at the developing brain barriers: ABC-transporters in the fetal and postnatal rat. Toxicol.Lett. 197 (2010) 51-59.

[19] M.E. Elsherbiny, A.O. El-Kadi, D.R. Brocks, The effect of beta-naphthoflavone on the metabolism of amiodarone by hepatic and extra-hepatic microsomes. Toxicol.Lett. 195 (2010) 147-154.

[20] S. Fa, D. Samardzija, L. Odzic, K. Pogrmic-Majkic, S. Kaisarevic, R. Kovacevic, N. Andric, Hexabromocyclododecane facilitates FSH activation of ERK1/2 and AKT through epidermal growth factor receptor in rat granulosa cells. Arch.Toxicol. 88 (2014) 345-354.

[21] G. Fan, C. Feng, F. Wu, W. Ye, F. Lin, C. Wang, J. Yan, G. Zhu, Y. Xiao, Y. Bi, Methionine choline reverses lead-induced cognitive and N-methyl-d-aspartate receptor subunit 1 deficits. Toxicology 272 (2010) 23-31.

[22] Y. Feng, J. Yin, Z. Jiao, J. Shi, M. Li, B. Shao, Bisphenol AF may cause testosterone reduction by directly affecting testis function in adult male rats. Toxicol.Lett. 211 (2012) 201-209.

[23] Y. Fujii, M. Kimura, Y. Ishii, R. Yamamoto, R. Morita, S.M. Hayashi, K. Suzuki, M. Shibutani, Effect of enzymatically modified isoquercitrin on preneoplastic liver cell lesions induced by thioacetamide promotion in a two-stage hepatocarcinogenesis model using rats. Toxicology 305 (2013) 30-40.

[24] M. Gahrs, R. Roos, P.L. Andersson, D. Schrenk, Role of the nuclear xenobiotic receptors CAR and PXR in induction of cytochromes P450 by non-dioxinlike polychlorinated biphenyls in cultured rat hepatocytes. Toxicol.Appl.Pharmacol. 272 (2013) 77-85.

[25] S. Ganesan, P. Bhattacharya, A.F. Keating, 7,12-Dimethylbenz[a]anthracene exposure induces the DNA repair response in neonatal rat ovaries. Toxicol.Appl.Pharmacol. 272 (2013) 690-696.

[26] D. Gentilcore, I. Porreca, F. Rizzo, E. Ganbaatar, E. Carchia, M. Mallardo, F.M. De, C. Ambrosino, Bisphenol A interferes with thyroid specific gene expression. Toxicology 304 (2013) 21-31.

[27] K. Goto, M. Imaoka, M. Goto, I. Kikuchi, T. Suzuki, T. Jindo, W. Takasaki, Effect of body-weight loading onto the articular cartilage on the occurrence of quinolone-induced chondrotoxicity in juvenile rats. Toxicol.Lett. 216 (2013) 124-129.

[28] X. Guo, Q. Meng, Q. Liu, C. Wang, H. Sun, J. Peng, X. Ma, T. Kaku, K. Liu, JBP485 improves gentamicin-induced acute renal failure by regulating the expression and function of Oat1 and Oat3 in rats. Toxicol.Appl.Pharmacol. 271 (2013) 285-295.

[29] T.L. Hectors, C. Vanparys, A. Pereira-Fernandes, D. Knapen, R. Blust, Mechanistic evaluation of the insulin response in H4IIE hepatoma cells: new endpoints for toxicity testing? Toxicol.Lett. 212 (2012) 180-189.

[30] W.Y. Hou, S.F. Xu, Q.N. Zhu, Y.F. Lu, X.G. Cheng, J. Liu, Age- and sex-related differences of organic anion-transporting polypeptide gene expression in livers of rats. Toxicol.Appl.Pharmacol. 280 (2014) 370-377.

[31] J. Hu, G. Du, W. Zhang, H. Huang, D. Chen, D. Wu, X. Wang, Short-term neonatal/prepubertal exposure of dibutyl phthalate (DBP) advanced pubertal timing and affected hypothalamic kisspeptin/GPR54 expression differently in female rats. Toxicology 314 (2013) 65-75.

[32] H. Huang, Z. He, C. Zhu, L. Liu, H. Kou, L. Shen, H. Wang, Prenatal ethanol exposure-induced adrenal developmental abnormality of male offspring rats and its possible intrauterine programming mechanisms. Toxicol.Appl.Pharmacol. 288 (2015) 84-94.

[33] L.H. Huang, J.Y. He, B.X. Yuan, Y.X. Cao, Lipid soluble smoke particles upregulate endothelin receptors in rat basilar artery. Toxicol.Lett. 197 (2010) 243-255.

[34] X.J. Huang, X. Wang, A. Ihsan, Q. Liu, X.J. Xue, S.J. Su, C.H. Yang, W. Zhou, Z.H. Yuan, Interactions of NADPH oxidase, renin-angiotensin-aldosterone system and reactive oxygen species in mequindox-mediated aldosterone secretion in Wistar rats. Toxicol.Lett. 198 (2010) 112-118.

[35] X. Jing, D. Ren, X. Wei, H. Shi, X. Zhang, R.G. Perez, H. Lou, H. Lou, Eriodictyol-7-O-glucoside activates Nrf2 and protects against cerebral ischemic injury. Toxicol.Appl.Pharmacol. 273 (2013) 672-679.

[36] E.M. Jung, B.S. An, K.C. Choi, E.B. Jeung, Potential estrogenic activity of triclosan in the uterus of immature rats and rat pituitary GH3 cells. Toxicol.Lett. 208 (2012) 142-148.

[37] A. Kakehashi, A. Hagiwara, N. Imai, K. Nagano, F. Nishimaki, M. Banton, M. Wei, S. Fukushima, H. Wanibuchi, Mode of action of ethyl tertiary-butyl ether hepatotumorigenicity in the rat: evidence for a role of oxidative stress via activation of CAR, PXR and PPAR signaling pathways. Toxicol.Appl.Pharmacol. 273 (2013) 390-400.

[38] M.S. Khan, H. Devaraj, N. Devaraj, Chrysin abrogates early hepatocarcinogenesis and induces apoptosis in N-nitrosodiethylamine-induced preneoplastic nodules in rats. Toxicol.Appl.Pharmacol. 251 (2011) 85-94.

[39] J.K. Kim, K.H. Jung, J.H. Noh, J.W. Eun, H.J. Bae, H.J. Xie, J.J. Jang, J.C. Ryu, W.S. Park, J.Y. Lee, S.W. Nam, Identification of characteristic molecular signature for volatile organic compounds in peripheral blood of rat. Toxicol.Appl.Pharmacol. 250 (2011) 162-169.

[40] J.Y. Kim, E.H. Han, H.G. Kim, K.N. Oh, S.K. Kim, K.Y. Lee, H.G. Jeong, Bisphenol A-induced aromatase activation is mediated by cyclooxygenase-2 up-regulation in rat testicular Leydig cells. Toxicol.Lett. 193 (2010) 200-208.

[41] J.C. Kwekel, A.L. Forgacs, K.J. Williams, T.R. Zacharewski, o-p'-DDT-mediated uterotrophy and gene expression in immature C57BL/6 mice and Sprague-Dawley rats. Toxicol.Appl.Pharmacol. 273 (2013) 532-541.

[42] T. Liu, Q. Meng, C. Wang, Q. Liu, X. Guo, H. Sun, J. Peng, X. Ma, T. Kaku, K. Liu, Changes in expression of renal Oat1, Oat3 and Mrp2 in cisplatin-induced acute renal failure after treatment of JBP485 in rats. Toxicol.Appl.Pharmacol. 264 (2012) 423-430.

[43] Y.J. Liu, Q. Gao, C.X. Wu, Z.Z. Guan, Alterations of nAChRs and ERK1/2 in the brains of rats with chronic fluorosis and their connections with the decreased capacity of learning and memory. Toxicol.Lett. 192 (2010) 324-329.

[44] C. Lopez-Granero, D. Cardona, E. Gimenez, R. Lozano, J. Barril, F. Sanchez-Santed, F. Canadas, Chronic dietary exposure to chlorpyrifos causes behavioral impairments, low activity of brain membrane-bound acetylcholinesterase, and increased brain acetylcholinesterase-R mRNA. Toxicology 308 (2013) 41-49.

[45] P. Loutchanwoot, P. Srivilai, H. Jarry, Effects of the natural endocrine disruptor equol on the pituitary function in adult male rats. Toxicology 304 (2013) 69-75.

[46] Z.F. Luo, B. Feng, J. Mu, W. Qi, W. Zeng, Y.H. Guo, Q. Pang, Z.L. Ye, L. Liu, F.H. Yuan, Effects of 4-phenylbutyric acid on the process and development of diabetic nephropathy induced in rats by streptozotocin: regulation of endoplasmic reticulum stress-oxidative activation. Toxicol.Appl.Pharmacol. 246 (2010) 49-57.

[47] H. Ma, J. Wang, S.Z. Abdel-Rahman, P.J. Boor, M.F. Khan, Induction of base excision repair enzymes NTH1 and APE1 in rat spleen following aniline exposure. Toxicol.Appl.Pharmacol. 267 (2013) 276-283.

[48] N. Ma, C.J. Nicholson, M. Wong, A.C. Holloway, D.B. Hardy, Fetal and neonatal exposure to nicotine leads to augmented hepatic and circulating triglycerides in adult male offspring due to increased expression of fatty acid synthase. Toxicol.Appl.Pharmacol. 275 (2014) 1-11.

[49] J.A. Madden, P.B. Hoyer, P.J. Devine, A.F. Keating, Acute 7,12-dimethylbenz[a]anthracene exposure causes differential concentration-dependent follicle depletion and gene expression in neonatal rat ovaries. Toxicol.Appl.Pharmacol. 276 (2014) 179-187.

[50] F.J. Moller, C. Ledwig, O. Zierau, T. Hertrampf, G.H. Degen, P. Diel, G. Vollmer, The rat prepubertal uterine myometrium and not the luminal epithelium is predominantly affected by a chronic dietary genistein exposure. Arch.Toxicol. 86 (2012) 1899-1910.

[51] R. Morita, K. Shimamoto, Y. Ishii, K. Kuwata, B. Ogawa, M. Imaoka, S.M. Hayashi, K. Suzuki, M. Shibutani, K. Mitsumori, Suppressive effect of enzymatically modified isoquercitrin on phenobarbital-induced liver tumor promotion in rats. Arch.Toxicol. 85 (2011) 1475-1484.

[52] D. Nakamura, Y. Yanagiba, Z. Duan, Y. Ito, A. Okamura, N. Asaeda, Y. Tagawa, C. Li, K. Taya, S.Y. Zhang, H. Naito, D.H. Ramdhan, M. Kamijima, T. Nakajima, Bisphenol A may cause testosterone reduction by adversely affecting both testis and pituitary systems similar to estradiol. Toxicol.Lett. 194 (2010) 16-25.

[53] R. Nault, S. Kim, T.R. Zacharewski, Comparison of TCDD-elicited genome-wide hepatic gene expression in Sprague-Dawley rats and C57BL/6 mice. Toxicol.Appl.Pharmacol. 267 (2013) 184-191.

[54] M. Osborne, M. Haltalli, R. Currie, J. Wright, N.J. Gooderham, Transdifferentiated rat pancreatic progenitor cells (AR42J-B13/H) respond to phenobarbital in a rat hepatocyte-specific manner. Toxicology 363-364 (2016) 10-18.

[55] L. Pan, X. Shi, S. Liu, X. Guo, M. Zhao, R. Cai, G. Sun, Fluoride promotes osteoblastic differentiation through canonical Wnt/beta-catenin signaling pathway. Toxicol.Lett. 225 (2014) 34-42.

[56] X. Pan, Y. Dai, X. Li, N. Niu, W. Li, F. Liu, Y. Zhao, Z. Yu, Inhibition of arsenic-induced rat liver injury by grape seed exact through suppression of NADPH oxidase and TGF-beta/Smad activation. Toxicol.Appl.Pharmacol. 254 (2011) 323-331.

[57] J. Ping, J.F. Wang, L. Liu, Y.E. Yan, F. Liu, Y.Y. Lei, H. Wang, Prenatal caffeine ingestion induces aberrant DNA methylation and histone acetylation of steroidogenic factor 1 and inhibits fetal adrenal steroidogenesis. Toxicology 321 (2014) 53-61.

[58] H. Pontes, P.G. de Pinho, E. Fernandes, P.S. Branco, L.M. Ferreira, H. Carmo, F. Remiao, F. Carvalho, M.L. Bastos, Metabolic interactions between ethanol and MDMA in primary cultured rat hepatocytes. Toxicology 270 (2010) 150-157.

[59] P. Rajesh, K. Balasubramanian, Gestational exposure to di(2-ethylhexyl) phthalate (DEHP) impairs pancreatic beta-cell function in F1 rat offspring. Toxicol.Lett. 232 (2015) 46-57.

[60] S. Ramm, A. Mally, Role of drug-independent stress factors in liver injury associated with diclofenac intake. Toxicology 312 (2013) 83-96.

[61] F. Rataj, F.J. Moller, M. Jahne, O. Zierau, P. Diel, G. Vollmer, G. Kretzschmar, Regulation of uterine AHR battery gene expression by 17beta-Estradiol is predominantly mediated by estrogen receptor alpha. Arch.Toxicol. 86 (2012) 1603-1612.

[62] J.M. Sanders, S.J. Coulter, G.A. Knudsen, J.K. Dunnick, G.E. Kissling, L.S. Birnbaum, Disruption of estrogen homeostasis as a mechanism for uterine toxicity in Wistar Han rats treated with tetrabromobisphenol A. Toxicol.Appl.Pharmacol. 298 (2016) 31-39.

[63] H. Sandhu, C.B. Xu, L. Edvinsson, Alteration in contractile G-protein coupled receptor expression by moist snuff and nicotine in rat cerebral arteries. Toxicol.Appl.Pharmacol. 252 (2011) 138-149.

[64] Q. Saquib, S.M. Attia, M.A. Siddiqui, M.A. Aboul-Soud, A.A. Al-Khedhairy, J.P. Giesy, J. Musarrat, Phorate-induced oxidative stress, DNA damage and transcriptional activation of p53 and caspase genes in male Wistar rats. Toxicol.Appl.Pharmacol. 259 (2012) 54-65.

[65] S. Sawada, A. Oberemm, T. Buhrke, J. Merschenz, A. Braeuning, A. Lampen, Proteomic analysis of 3-MCPD and 3-MCPD dipalmitate-induced toxicity in rat kidney. Arch.Toxicol. 90 (2016) 1437-1448.

[66] H. Shen, L.W. Robertson, G. Ludewig, Regulation of paraoxonase 1 (PON1) in PCB 126-exposed male Sprague Dawley rats. Toxicol.Lett. 209 (2012) 291-298.

[67] Y.Q. Shi, Y.P. Wang, Y. Song, H.W. Li, C.J. Liu, Z.G. Wu, K.D. Yang, p,p'-DDE induces testicular apoptosis in prepubertal rats via the Fas/FasL pathway. Toxicol.Lett. 193 (2010) 79-85.

[68] Z. Shi, H. Zhang, L. Ding, Y. Feng, J. Wang, J. Dai, Proteomic analysis for testis of rats chronically exposed to perfluorododecanoic acid. Toxicol.Lett. 192 (2010) 179-188.

[69] Y. Shimada, Y. Dewa, R. Ichimura, T. Suzuki, S. Mizukami, S.M. Hayashi, M. Shibutani, K. Mitsumori, Antioxidant enzymatically modified isoquercitrin suppresses the development of liver preneoplastic lesions in rats induced by beta-naphthoflavone. Toxicology 268 (2010) 213-218.

[70] K. Shimamoto, Y. Dewa, S. Kemmochi, E. Taniai, H. Hayashi, M. Imaoka, M. Shibutani, K. Mitsumori, Relationship between CYP1A induction by indole-3-carbinol or flutamide and liver tumor-promoting potential in rats. Arch.Toxicol. 85 (2011) 1159-1166.

[71] A. Shiraki, H. Akane, T. Ohishi, L. Wang, R. Morita, K. Suzuki, K. Mitsumori, M. Shibutani, Similar distribution changes of GABAergic interneuron subpopulations in contrast to the different impact on neurogenesis between developmental and adult-stage hypothyroidism in the hippocampal dentate gyrus in rats. Arch.Toxicol. 86 (2012) 1559-1569.

[72] K.A. Solak, F.M. Wijnolts, F.P. Pralong, B.J. Blaauboer, M. van den Berg, R.H. Westerink, M.B. van Duursen, In vitro neuroendocrine effects of 2,3,7,8-tetrachlorodibenzo-p-dioxin (TCDD) in the AhR-expressing hypothalamic rat GnV-3 cell line. Toxicology 311 (2013) 124-134.

[73] B. Sosic-Jurjevic, B. Filipovic, E.K. Wirth, J. Zivanovic, N. Radulovic, S. Jankovic, V. Milosevic, J. Kohrle, Soy isoflavones interfere with thyroid hormone homeostasis in orchidectomized middle-aged rats. Toxicol.Appl.Pharmacol. 278 (2014) 124-134.

[74] M. Stamou, E. Uwimana, B.M. Flannery, I. Kania-Korwel, H.J. Lehmler, P.J. Lein, Subacute nicotine co-exposure has no effect on 2,2',3,5',6- pentachlorobiphenyl disposition but alters hepatic cytochrome P450 expression in the male rat. Toxicology 338 (2015) 59-68.

[75] M. Stephanie, P. Carlos, A. Seyfried, M. Piechotta, P. Diel, The anabolic steroid methandienone targets the hypothalamic–pituitary–testicular axis and myostatin signaling in a rat training model. Arch.Toxicol. 86 (2012) 109-119.

[76] T. Suzuki, M. Jin, Y. Dewa, R. Ichimura, Y. Shimada, S. Mizukami, M. Shibutani, K. Mitsumori, Evaluation of in vivo liver genotoxic potential of Wy-14,643 and piperonyl butoxide in rats subjected to two-week repeated oral administration. Arch.Toxicol. 84 (2010) 493-500.

[77] T. Tanaka, S. Mizukami, Y. Hasegawa-Baba, N. Onda, Y. Sugita-Konishi, T. Yoshida, M. Shibutani, Developmental exposure of aflatoxin B1 reversibly affects hippocampal neurogenesis targeting late-stage neural progenitor cells through suppression of cholinergic signaling in rats. Toxicology 336 (2015) 59-69.

[78] E. Taniai, A. Yafune, M. Nakajima, S.M. Hayashi, F. Nakane, M. Itahashi, M. Shibutani, Ochratoxin A induces karyomegaly and cell cycle aberrations in renal tubular cells without relation to induction of oxidative stress responses in rats. Toxicol.Lett. 224 (2014) 64-72.

[79] M.M. Tawfeeq, T. Suzuki, K. Shimamoto, H. Hayashi, M. Shibutani, K. Mitsumori, Evaluation of in vivo genotoxic potential of fenofibrate in rats subjected to two-week repeated oral administration. Arch.Toxicol. 85 (2011) 1003-1011.

[80] K.I. van Ede, P.L. Andersson, K.P. Gaisch, M. van den Berg, M.B. van Duursen, Comparison of intake and systemic relative effect potencies of dioxin-like compounds in female rats after a single oral dose. Arch.Toxicol. 88 (2014) 637-646.

[81] B.D. van, C. Albrecht, A.M. Knaapen, F.R. Cassee, M.E. Gerlofs-Nijland, I.M. Kooter, N. Palomero-Gallagher, H.J. Bidmon, F.J. van Schooten, J. Krutmann, R.P. Schins, Comparative evaluation of the effects of short-term inhalation exposure to diesel engine exhaust on rat lung and brain. Arch.Toxicol. 84 (2010) 553-562.

[82] M.W. Walters, K.B. Wallace, Urea cycle gene expression is suppressed by PFOA treatment in rats. Toxicol.Lett. 197 (2010) 46-50.

[83] J. Wang, G. Wang, H. Ma, M.F. Khan, Enhanced expression of cyclins and cyclin-dependent kinases in aniline-induced cell proliferation in rat spleen. Toxicol.Appl.Pharmacol. 250 (2011) 213-220.

[84] Z. Wei, L. Song, J. Wei, T. Chen, J. Chen, Y. Lin, W. Xia, B. Xu, X. Li, X. Chen, Y. Li, S. Xu, Maternal exposure to di-(2-ethylhexyl)phthalate alters kidney development through the renin-angiotensin system in offspring. Toxicol.Lett. 212 (2012) 212-221.

[85] J. Wu, T. Yang, X. Li, Q. Yang, R. Liu, J. Huang, Y. Li, C. Yang, Y. Jiang, Alteration of serum miR-206 and miR-133b is associated with lung carcinogenesis induced by 4-(methylnitrosamino)-1-(3-pyridyl)-1-butanone. Toxicol.Appl.Pharmacol. 267 (2013) 238-246.

[86] D. Xu, G. Liang, Y.E. Yan, W.W. He, Y.S. Liu, L.B. Chen, J. Magdalou, H. Wang, Nicotine-induced over-exposure to maternal glucocorticoid and activated glucocorticoid metabolism causes hypothalamic-pituitary-adrenal axis-associated neuroendocrine metabolic alterations in fetal rats. Toxicol.Lett. 209 (2012) 282-290.

[87] X. Yan, X. Yan, A. Morrison, T. Han, Q. Chen, J. Li, J. Wang, Fluoride induces apoptosis and alters collagen I expression in rat osteoblasts. Toxicol.Lett. 200 (2011) 133-138.

[88] Y.E. Yan, L. Liu, J.F. Wang, F. Liu, X.H. Li, H.Q. Qin, H. Wang, Prenatal nicotinic exposure suppresses fetal adrenal steroidogenesis via steroidogenic factor 1 (SF-1) deacetylation. Toxicol.Appl.Pharmacol. 277 (2014) 231-241.

[89] L. Ye, B. Zhao, K. Yuan, Y. Chu, C. Li, C. Zhao, Q.Q. Lian, R.S. Ge, Gene expression profiling in fetal rat lung during gestational perfluorooctane sulfonate exposure. Toxicol.Lett. 209 (2012) 270-276.

[90] W.G. Yu, W. Liu, L. Liu, Y.H. Jin, Perfluorooctane sulfonate increased hepatic expression of OAPT2 and MRP2 in rats. Arch.Toxicol. 85 (2011) 613-621.

[91] D.S. Zhang, Z.Y. Liu, Y.J. Li, Z.L. Sun, NQO1 involves in the imine bond reduction of sanguinarine and recombinant adeno-associated virus mediated NQO1 overexpression decreases sanguinarine-induced cytotoxicity in rat BRL cells. Toxicol.Lett. 225 (2014) 119-129.

[92] H.Y. Zhang, W.Y. Xue, Y.Y. Li, Y. Ma, Y.S. Zhu, W.Q. Huo, B. Xu, W. Xia, S.Q. Xu, Perinatal exposure to 4-nonylphenol affects adipogenesis in first and second generation rats offspring. Toxicol.Lett. 225 (2014) 325-332.

[93] L. Zhang, T. Nagai, K. Yamada, D. Ibi, S. Ichihara, K. Subramanian, Z. Huang, S.S. Mohideen, H. Naito, G. Ichihara, Effects of sub-acute and sub-chronic inhalation of 1-bromopropane on neurogenesis in adult rats. Toxicology 304 (2013) 76-82.

[94] Q. Zhang, P. Zou, H. Zhan, M. Zhang, L. Zhang, R.S. Ge, Y. Huang, Dihydrolipoamide dehydrogenase and cAMP are associated with cadmium-mediated Leydig cell damage. Toxicol.Lett. 205 (2011) 183-189.

[95] W. Zhang, X.J. Li, X. Zeng, D.Y. Shen, C.Q. Liu, H.J. Zhang, C.B. Xu, X.Y. Li, Activation of nuclear factor-kappaB pathway is responsible for tumor necrosis factor-alpha-induced up-regulation of endothelin B2 receptor expression in vascular smooth muscle cells in vitro. Toxicol.Lett. 209 (2012) 107-112.

[96] Z. Zhang, Z.Z. Sun, X. Xiao, S. Zhou, X.C. Wang, J. Gu, L.L. Qiu, X.H. Zhang, Q. Xu, B. Zhen, X. Wang, S.L. Wang, Mechanism of BDE209-induced impaired glucose homeostasis based on gene microarray analysis of adult rat liver. Arch.Toxicol. 87 (2013) 1557-1567.

[97] H. Zhao, S. Xu, Z. Wang, Y. Li, W. Guo, C. Lin, S. Gong, C. Li, G. Wang, L. Cai, Repetitive exposures to low-dose X-rays attenuate testicular apoptotic cell death in streptozotocin-induced diabetes rats. Toxicol.Lett. 192 (2010) 356-364.

[98] J. Zhao, Y. Xie, C. Qian, L. Li, R. Jiang, H. Kan, R. Chen, W. Song, Imbalance of Th1 and Th2 cells in cardiac injury induced by ambient fine particles. Toxicol.Lett. 208 (2012) 225-231.

[99] L. Zheng, J. Yang, Q. Liu, F. Yu, S. Wu, C. Jin, X. Lu, L. Zhang, Y. Du, Q. Xi, Y. Cai, Lanthanum chloride impairs spatial learning and memory and downregulates NF-kappaB signalling pathway in rats. Arch.Toxicol. 87 (2013) 2105-2117.

[100] B.N. Zordoky, A. Anwar-Mohamed, M.E. Aboutabl, A.O. El-Kadi, Acute doxorubicin cardiotoxicity alters cardiac cytochrome P450 expression and arachidonic acid metabolism in rats. Toxicol.Appl.Pharmacol. 242 (2010) 38-46.
